# (4-Fluoro­phen­yl)[8-(4-fluoro­benzo­yl)-2,7-diphen­oxy­naphthalen-1-yl]methan­one

**DOI:** 10.1107/S160053681204408X

**Published:** 2012-10-31

**Authors:** Daichi Hijikata, Kosuke Sasagawa, Sayaka Yoshiwaka, Akiko Okamoto, Noriyuki Yonezawa

**Affiliations:** a Department of Organic and Polymer Materials Chemistry, Tokyo University of Agriculture & Technology (TUAT), Koganei, Tokyo 184.8588, Japan

## Abstract

In the title compound, C_36_H_22_F_2_O_4_, the aromatic rings of the benzoyl and phen­oxy groups make dihedral angles of 72.07 (5), 73.24 (5), 62.49 (5) and 77.96 (6)° with the naphthalene ring system. In the crystal, C—H⋯O hydrogen bonds and C—H⋯π inter­actions are observed.

## Related literature

For information on electrophilic aromatic aroylation of the naphthalene core, see: Okamoto & Yonezawa (2009 ▶); Okamoto *et al.* (2011 ▶, 2012 ▶). For the structures of closely related compounds, see: Watanabe *et al.* (2010 ▶); Sakamoto *et al.* (2012 ▶).
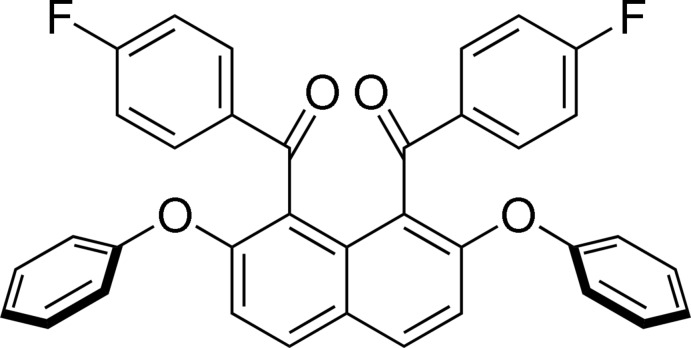


## Experimental

### 

#### Crystal data

C_36_H_22_F_2_O_4_
*M* *_r_* = 556.54
Orthorhombic, 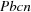

*a* = 22.3058 (4) Å
*b* = 14.6047 (3) Å
*c* = 16.8302 (3) Å
*V* = 5482.76 (18) Å^3^
*Z* = 8
Cu *K*α radiation
μ = 0.80 mm^−1^
*T* = 193 K
0.50 × 0.30 × 0.10 mm

#### Data collection

Rigaku R-AXIS RAPID diffractometer
Absorption correction: numerical (*NUMABS*; Higashi, 1999 ▶) *T*_min_ = 0.691, *T*_max_ = 0.925
96215 measured reflections
5011 independent reflections
4671 reflections with *I* > 2σ(*I*)
*R*_int_ = 0.016

#### Refinement

*R*[*F* ^2^ > 2σ(*F* ^2^)] = 0.035
*wR*(*F* ^2^) = 0.091
*S* = 1.06
5011 reflections
380 parameters
H-atom parameters constrained
Δρ_max_ = 0.17 e Å^−3^
Δρ_min_ = −0.16 e Å^−3^

### 

Data collection: *PROCESS-AUTO* (Rigaku, 1998 ▶); cell refinement: *PROCESS-AUTO*; data reduction: *PROCESS-AUTO*; program(s) used to solve structure: *Il Milione* (Burla *et al.*, 2007 ▶); program(s) used to refine structure: *SHELXL97* (Sheldrick, 2008 ▶); molecular graphics: *ORTEPIII* (Burnett & Johnson, 1996 ▶); software used to prepare material for publication: *SHELXL97*.

## Supplementary Material

Crystal structure: contains datablock(s) I, global. DOI: 10.1107/S160053681204408X/rz5018sup1.cif

Structure factors: contains datablock(s) I. DOI: 10.1107/S160053681204408X/rz5018Isup2.hkl

Supplementary material file. DOI: 10.1107/S160053681204408X/rz5018Isup3.cml

Additional supplementary materials:  crystallographic information; 3D view; checkCIF report

## Figures and Tables

**Table 1:** Hydrogen-bond geometry (Å, °) *Cg* is the centroid of the C31–C36 ring.

*D*—H⋯*A*	*D*—H	H⋯*A*	*D*⋯*A*	*D*—H⋯*A*
C6—H6⋯O3^i^	0.95	2.40	3.3477 (15)	172
C13—H13⋯*Cg* ^ii^	0.95	2.87	3.6924 (15)	145

Symmetry codes: (i) 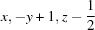; (ii) 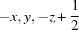.

**Fig. 1.**
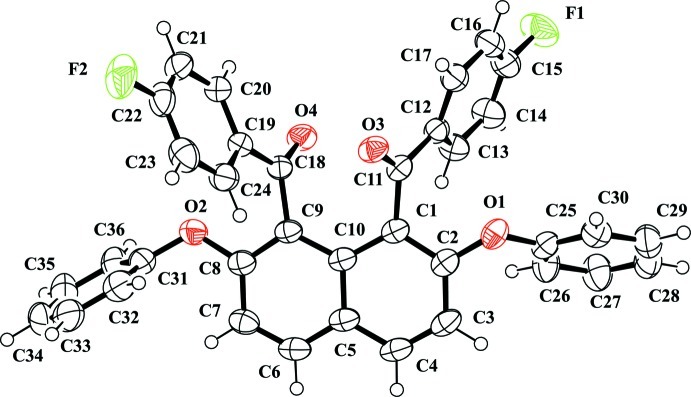
The molecular structure of title compound, showing 50% probability displacement ellipsoids.

**Fig. 2.**
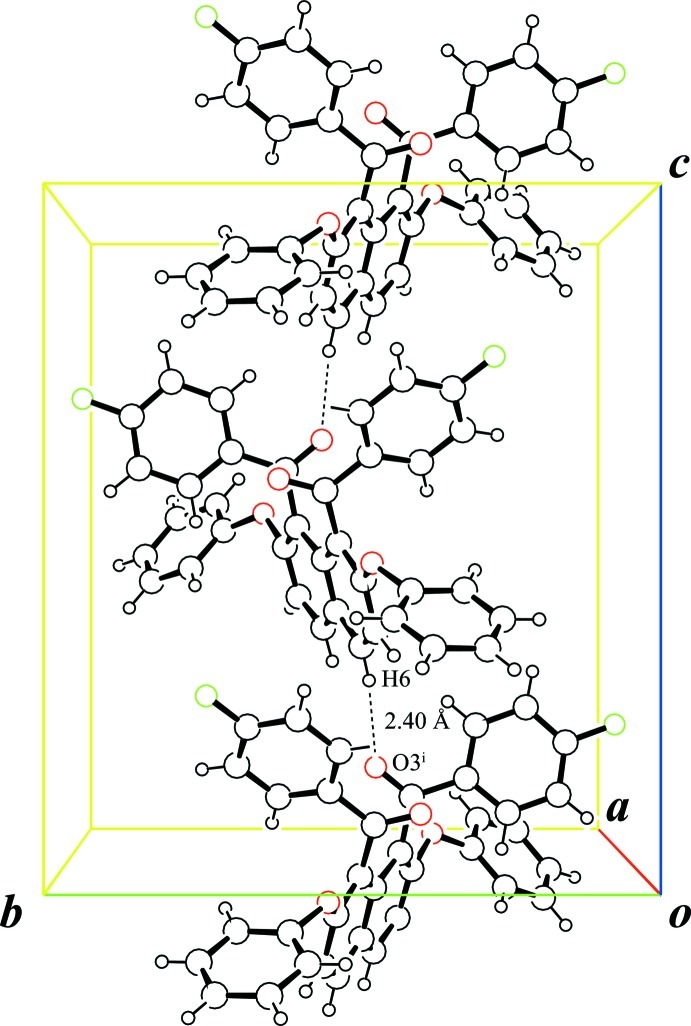
A partial crystal packing diagram of title compound. The intermolecular C—H···O interactions are shown as dashed lines.

**Fig. 3.**
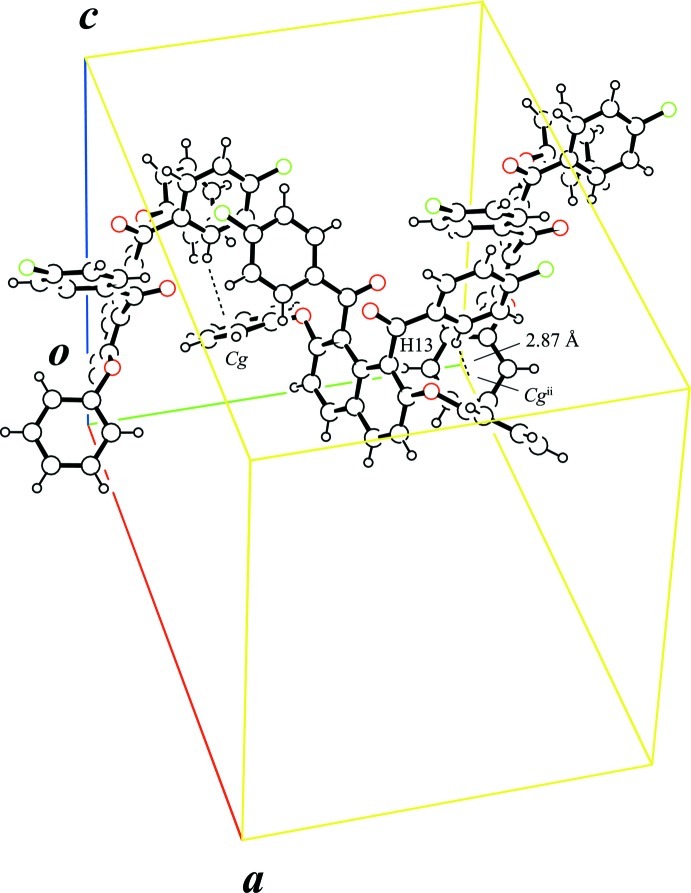
A partial crystal packing diagram of title compound. The intermolecular C—H···π interactions are shown as dashed lines.

## supplementary crystallographic information

### Comment

In the course of our study on electrophilic aromatic aroylation of 2,7-dimethoxynaphthalene, *peri*-aroylnaphthalene compounds have proven to be formed regioselectively with the aid of suitable acidic mediators (Okamoto & Yonezawa, 2009; Okamoto *et al.*, 2011). As one of applications, the authors have integrated the resulting molecular unit to poly(ether ketone) backbone *via* nucleophilic aromatic substitution polycondensation (Okamoto *et al.*, 2012). The poly(ether ketone)s composed of 1,8-diaroylenenaphthalene units show unique thermal properties and solubility for organic solvents. These curious features of the polymers can be explained on the basis of structural features of the 1,8-diaroylene naphthalene units. Under these circumstances, the authors have stimulated the X-ray crystal structural study of several 1,8-diaroylated naphthalene analogues exemplified by (2,7-dimethoxynaphthalene-1,8-diyl)bis(4-fluorophenyl)dimethanone (Watanabe *et al.*, 2010) and 1,8-dibenzoylnaphthalene-2,7-diyl dibenzoate (Sakamoto *et al.*, 2012). These molecules have essentially the same non-coplanar features. The aroyl groups at the 1,8-positions of the naphthalene rings in these molecules are twistedly bonded in an almost perpendicular fashion, but the benzene ring moieties of the aroyl groups tilt slightly toward the *exo* sides of the naphthalene rings. As a part of our continuous study on the molecular structures of this kind of homologous molecules, the X-ray crystal structure of title compound, 1,8-bis(4-fluorobenoyl)-2,7-diphenoxynaphthalene, is discussed in this article.

The molecular structure of the title compound is displayed in Fig. 1. Two benzoyl groups at 1,8-positions of the naphthalene ring are situated in opposite directions, anti orientation. The benzene rings of the benzoyl groups make dihedral angles of 72.07 (5) and 73.24 (5)° with the naphthalene ring, respectively. The dihedral angles between the phenyl rings of phenoxy groups and the naphthalene ring system are 62.49 (5) and 77.96 (6)°, respectively. The crystal packing is stabilized by an intermolecular C—H···O hydrogen bond between the oxygen atom (O3) of the carbonyl group of the adjacent molecule and one hydrogen atom (H6) on the naphthalene ring along the *c* axis (C6—H6···O3^i^ = 2.40 Å; Fig. 2 and Table 1). The C—H···π interaction (C13—H13···*Cg*^ii^ = 2.87 Å; Fig. 3 and Table 1) also contributes to the stabilization of the aromatic ring alignments and the crystal structure.

### Experimental

To a 10 ml flask, 1,8-bis(4-fluorobenzoyl)-2,7-dihydroxynaphthalene (1.0 mmol, 404 mg), benzeneboronic acid (4.0 mmol, 487 mg), Cu(OAc)_2_ (2.0 mmol, 363 mg), activated 4 Å molecular sieves (1.0 g), pyridine (8.0 mmol, 632 mg), methylene chloride (4.0 ml) were placed. The reaction mixture was stirred at room temperature for 48 h. After the reaction, the mixture was extracted with CHCl_3_. The combined extracts were washed with saturated NH_4_Cl_aq_ and 2*M* aqueous HCl followed by washing with brine. The organic layers thus obtained were dried over anhydrous MgSO_4_. The solvent was removed under reduced pressure to give cake. The crude product was purified by column chromatography (silica gel, hexane/CHCl_3_, 1:1 *v*/*v*) to give the title compound (isolated yield 46%). Furthermore, the isolated product was crystallized from methanol to give single crystal.

^1^HNMR δ (300 MHz, CDCl_3_): 6.81(4*H*, d, *J*=7.5 Hz), 7.01(4*H*, t, *J*=8.5 Hz), 7.05(2*H*, t, *J*=7.5 Hz), 7.08(2*H*, d, *J*=8.9 Hz), 7.22(4*H*, t, *J*=7.5 Hz), 7.81(4*H*, dd, *J*=8.5, 5.5 Hz), 7.90(2*H*, d, *J*=8.9 Hz) p.p.m..

^13^CNMR δ (75 MHz, CDCl_3_): 115.18(d, ^2^*J*_C—F_=22.4 Hz), 117.21, 118.92, 123.92, 124.65, 127.85, 129.69, 130.65, 131.76(d, ^3^*J*_C—F_=10.1 Hz), 132.12, 134.87(d, ^4^*J*_C—F_=2.1 Hz), 153.73, 155.79, 165.64(d, ^1^*J*_C—F_=255.7 Hz), 194.64 p.p.m..

IR (KBr): 1666 (C=O), 1594, 1504, 1486 (Ar, naphthalene), 1262 (=C—O—C) cm^-1^.

HRMS (m/*z*): [*M* + H]^+^ calcd for C_36_H_23_F_2_O_4_, 557.1564 found, 557.1569.

m.p. 441.9–443.6 K

### Refinement

All H atoms were put in calculated positions and treated as riding on their parent atoms, with C—H = 0.95 Å, and *U*_iso_(H) = 1.2 *U*_eq_(C).

### Crystal data

**Table:**

C_36_H_22_F_2_O_4_	*F*(000) = 2304
*M**_r_* = 556.54	*D*_x_ = 1.348 Mg m^−^^3^
Orthorhombic, *P**b**c**n*	Cu *K*α radiation, λ = 1.54187 Å
Hall symbol: -P 2n 2ab	Cell parameters from 89526 reflections
*a* = 22.3058 (4) Å	θ = 3.0–68.2°
*b* = 14.6047 (3) Å	µ = 0.80 mm^−^^1^
*c* = 16.8302 (3) Å	*T* = 193 K
*V* = 5482.76 (18) Å^3^	Block, colorless
*Z* = 8	0.50 × 0.30 × 0.10 mm

### Data collection

**Table:**

Rigaku R-AXIS RAPID diffractometer	5011 independent reflections
Radiation source: rotating anode	4671 reflections with *I* > 2σ(*I*)
Graphite monochromator	*R*_int_ = 0.016
Detector resolution: 10.000 pixels mm^-1^	θ_max_ = 68.2°, θ_min_ = 3.6°
ω scans	*h* = −26→26
Absorption correction: numerical (*NUMABS*; Higashi, 1999)	*k* = −17→17
*T*_min_ = 0.691, *T*_max_ = 0.925	*l* = −19→19
96215 measured reflections	

### Refinement

**Table:**

Refinement on *F*^2^	Secondary atom site location: difference Fourier map
Least-squares matrix: full	Hydrogen site location: inferred from neighbouring sites
*R*[*F*^2^ > 2σ(*F*^2^)] = 0.035	H-atom parameters constrained
*wR*(*F*^2^) = 0.091	*w* = 1/[σ^2^(*F*_o_^2^) + (0.0435*P*)^2^ + 1.3362*P*] where *P* = (*F*_o_^2^ + 2*F*_c_^2^)/3
*S* = 1.06	(Δ/σ)_max_ < 0.001
5011 reflections	Δρ_max_ = 0.17 e Å^−^^3^
380 parameters	Δρ_min_ = −0.16 e Å^−^^3^
0 restraints	Extinction correction: *SHELXL97* (Sheldrick, 2008), Fc^*^=kFc[1+0.001xFc^2^λ^3^/sin(2θ)]^-1/4^
Primary atom site location: structure-invariant direct methods	Extinction coefficient: 0.00098 (6)

### Special details

**Table:**

Geometry. All e.s.d.'s (except the e.s.d. in the dihedral angle between two l.s. planes) are estimated using the full covariance matrix. The cell e.s.d.'s are taken into account individually in the estimation of e.s.d.'s in distances, angles and torsion angles; correlations between e.s.d.'s in cell parameters are only used when they are defined by crystal symmetry. An approximate (isotropic) treatment of cell e.s.d.'s is used for estimating e.s.d.'s involving l.s. planes.
Refinement. Refinement of *F*^2^ against ALL reflections. The weighted *R*-factor *wR* and goodness of fit *S* are based on *F*^2^, conventional *R*-factors *R* are based on *F*, with *F* set to zero for negative *F*^2^. The threshold expression of *F*^2^ > σ(*F*^2^) is used only for calculating *R*-factors(gt) *etc*. and is not relevant to the choice of reflections for refinement. *R*-factors based on *F*^2^ are statistically about twice as large as those based on *F*, and *R*- factors based on ALL data will be even larger.

### Fractional atomic coordinates and isotropic or equivalent isotropic displacement parameters (Å^2^)

**Table:**

	*x*	*y*	*z*	*U*_iso_*/*U*_eq_	
F1	0.42732 (5)	0.97093 (6)	0.71349 (6)	0.0794 (3)	
F2	0.21941 (5)	0.25563 (7)	0.76820 (6)	0.0814 (3)	
O1	0.51813 (4)	0.65226 (6)	0.53754 (5)	0.0445 (2)	
O2	0.22348 (4)	0.46286 (6)	0.46362 (5)	0.0476 (2)	
O3	0.40405 (4)	0.54759 (6)	0.64967 (5)	0.0436 (2)	
O4	0.28295 (4)	0.62180 (6)	0.58844 (6)	0.0485 (2)	
C1	0.42155 (5)	0.59134 (7)	0.51684 (7)	0.0354 (3)	
C2	0.47700 (5)	0.61326 (8)	0.48603 (7)	0.0392 (3)	
C3	0.49455 (6)	0.58889 (8)	0.40848 (8)	0.0452 (3)	
H3	0.5336	0.6032	0.3895	0.054*	
C4	0.45474 (6)	0.54468 (8)	0.36146 (8)	0.0450 (3)	
H4	0.4662	0.5284	0.3090	0.054*	
C5	0.39650 (6)	0.52212 (7)	0.38826 (7)	0.0393 (3)	
C6	0.35582 (6)	0.47834 (8)	0.33668 (7)	0.0437 (3)	
H6	0.3679	0.4649	0.2838	0.052*	
C7	0.29964 (6)	0.45476 (8)	0.36058 (7)	0.0430 (3)	
H7	0.2729	0.4244	0.3254	0.052*	
C8	0.28201 (5)	0.47632 (8)	0.43851 (7)	0.0389 (3)	
C9	0.31971 (5)	0.51855 (7)	0.49203 (7)	0.0353 (3)	
C10	0.37908 (5)	0.54365 (7)	0.46804 (7)	0.0351 (3)	
C11	0.41193 (5)	0.61111 (8)	0.60379 (7)	0.0362 (3)	
C12	0.41448 (5)	0.70744 (8)	0.63178 (7)	0.0380 (3)	
C13	0.40891 (6)	0.78060 (9)	0.57966 (8)	0.0460 (3)	
H13	0.4022	0.7693	0.5248	0.055*	
C14	0.41308 (7)	0.87001 (9)	0.60688 (9)	0.0549 (3)	
H14	0.4093	0.9203	0.5714	0.066*	
C15	0.42273 (7)	0.88356 (9)	0.68609 (9)	0.0542 (3)	
C16	0.42761 (7)	0.81373 (10)	0.73981 (8)	0.0536 (3)	
H16	0.4338	0.8260	0.7946	0.064*	
C17	0.42336 (6)	0.72486 (9)	0.71226 (8)	0.0458 (3)	
H17	0.4265	0.6752	0.7485	0.055*	
C18	0.29239 (5)	0.54231 (8)	0.57138 (7)	0.0364 (3)	
C19	0.27487 (5)	0.46544 (8)	0.62434 (7)	0.0380 (3)	
C20	0.23468 (6)	0.48195 (10)	0.68603 (7)	0.0466 (3)	
H20	0.2197	0.5420	0.6946	0.056*	
C21	0.21664 (6)	0.41109 (11)	0.73482 (8)	0.0576 (4)	
H21	0.1895	0.4219	0.7773	0.069*	
C22	0.23845 (7)	0.32519 (11)	0.72109 (8)	0.0564 (4)	
C23	0.27865 (7)	0.30606 (10)	0.66188 (9)	0.0555 (4)	
H23	0.2936	0.2457	0.6544	0.067*	
C24	0.29691 (6)	0.37735 (9)	0.61327 (8)	0.0469 (3)	
H24	0.3248	0.3659	0.5718	0.056*	
C25	0.55286 (5)	0.72530 (8)	0.51134 (8)	0.0413 (3)	
C26	0.53493 (6)	0.78388 (9)	0.45175 (9)	0.0541 (3)	
H26	0.4978	0.7747	0.4254	0.065*	
C27	0.57176 (7)	0.85648 (10)	0.43073 (10)	0.0621 (4)	
H27	0.5600	0.8965	0.3890	0.075*	
C28	0.62528 (7)	0.87125 (10)	0.46961 (10)	0.0591 (4)	
H28	0.6504	0.9209	0.4548	0.071*	
C29	0.64195 (6)	0.81319 (10)	0.53019 (10)	0.0562 (4)	
H29	0.6784	0.8238	0.5578	0.067*	
C30	0.60613 (6)	0.73941 (9)	0.55138 (8)	0.0473 (3)	
H30	0.6181	0.6992	0.5928	0.057*	
C31	0.18912 (6)	0.39816 (9)	0.42294 (7)	0.0441 (3)	
C32	0.20515 (7)	0.30699 (10)	0.42398 (9)	0.0570 (4)	
H32	0.2393	0.2873	0.4530	0.068*	
C33	0.17099 (8)	0.24501 (11)	0.38237 (11)	0.0675 (4)	
H33	0.1820	0.1822	0.3819	0.081*	
C34	0.12115 (9)	0.27327 (12)	0.34162 (10)	0.0717 (5)	
H34	0.0982	0.2302	0.3123	0.086*	
C35	0.10419 (8)	0.36443 (12)	0.34296 (10)	0.0678 (4)	
H35	0.0690	0.3835	0.3158	0.081*	
C36	0.13857 (6)	0.42802 (10)	0.38408 (8)	0.0524 (3)	
H36	0.1274	0.4908	0.3853	0.063*	

### Atomic displacement parameters (Å^2^)

**Table:**

	*U*^11^	*U*^22^	*U*^33^	*U*^12^	*U*^13^	*U*^23^
F1	0.1211 (8)	0.0418 (5)	0.0751 (6)	−0.0105 (5)	0.0047 (6)	−0.0162 (4)
F2	0.0877 (7)	0.0902 (7)	0.0662 (6)	−0.0393 (6)	−0.0098 (5)	0.0362 (5)
O1	0.0405 (4)	0.0453 (5)	0.0476 (5)	−0.0009 (4)	0.0052 (4)	0.0125 (4)
O2	0.0421 (5)	0.0541 (5)	0.0467 (5)	0.0010 (4)	0.0022 (4)	−0.0123 (4)
O3	0.0529 (5)	0.0386 (4)	0.0393 (4)	0.0002 (4)	0.0101 (4)	0.0055 (4)
O4	0.0537 (5)	0.0363 (5)	0.0555 (6)	0.0036 (4)	0.0137 (4)	−0.0079 (4)
C1	0.0413 (6)	0.0277 (5)	0.0371 (6)	0.0062 (4)	0.0077 (5)	0.0052 (4)
C2	0.0423 (6)	0.0309 (5)	0.0444 (7)	0.0045 (5)	0.0068 (5)	0.0083 (5)
C3	0.0478 (7)	0.0369 (6)	0.0510 (7)	0.0052 (5)	0.0200 (6)	0.0087 (5)
C4	0.0601 (8)	0.0349 (6)	0.0399 (6)	0.0075 (5)	0.0196 (6)	0.0039 (5)
C5	0.0534 (7)	0.0285 (5)	0.0360 (6)	0.0081 (5)	0.0114 (5)	0.0038 (4)
C6	0.0649 (8)	0.0339 (6)	0.0322 (6)	0.0077 (5)	0.0092 (5)	0.0010 (5)
C7	0.0567 (7)	0.0358 (6)	0.0363 (6)	0.0049 (5)	0.0000 (5)	−0.0010 (5)
C8	0.0446 (6)	0.0335 (6)	0.0387 (6)	0.0052 (5)	0.0038 (5)	0.0010 (5)
C9	0.0428 (6)	0.0292 (5)	0.0339 (6)	0.0055 (4)	0.0059 (5)	0.0014 (4)
C10	0.0447 (6)	0.0257 (5)	0.0350 (6)	0.0063 (4)	0.0080 (5)	0.0042 (4)
C11	0.0336 (5)	0.0358 (6)	0.0391 (6)	0.0021 (4)	0.0044 (5)	0.0036 (5)
C12	0.0359 (6)	0.0379 (6)	0.0402 (6)	0.0000 (5)	0.0042 (5)	−0.0003 (5)
C13	0.0555 (7)	0.0394 (6)	0.0432 (7)	0.0061 (5)	−0.0017 (6)	−0.0006 (5)
C14	0.0733 (9)	0.0370 (7)	0.0545 (8)	0.0054 (6)	−0.0004 (7)	0.0020 (6)
C15	0.0654 (9)	0.0373 (7)	0.0598 (9)	−0.0044 (6)	0.0045 (7)	−0.0093 (6)
C16	0.0637 (9)	0.0530 (8)	0.0442 (7)	−0.0099 (7)	0.0016 (6)	−0.0088 (6)
C17	0.0513 (7)	0.0443 (7)	0.0418 (7)	−0.0059 (6)	0.0023 (5)	0.0020 (5)
C18	0.0348 (6)	0.0359 (6)	0.0386 (6)	0.0016 (4)	0.0039 (5)	−0.0057 (5)
C19	0.0393 (6)	0.0412 (6)	0.0334 (6)	−0.0057 (5)	0.0017 (5)	−0.0046 (5)
C20	0.0448 (7)	0.0563 (8)	0.0388 (6)	−0.0088 (6)	0.0051 (5)	−0.0083 (6)
C21	0.0541 (8)	0.0796 (11)	0.0391 (7)	−0.0206 (7)	0.0078 (6)	0.0003 (7)
C22	0.0619 (8)	0.0653 (9)	0.0419 (7)	−0.0281 (7)	−0.0080 (6)	0.0135 (6)
C23	0.0700 (9)	0.0433 (7)	0.0532 (8)	−0.0095 (6)	−0.0081 (7)	0.0063 (6)
C24	0.0571 (8)	0.0416 (7)	0.0420 (7)	−0.0025 (6)	0.0040 (6)	−0.0008 (5)
C25	0.0392 (6)	0.0385 (6)	0.0462 (7)	0.0029 (5)	0.0081 (5)	0.0035 (5)
C26	0.0507 (7)	0.0469 (7)	0.0646 (9)	−0.0070 (6)	−0.0085 (6)	0.0148 (6)
C27	0.0709 (10)	0.0485 (8)	0.0670 (10)	−0.0130 (7)	−0.0058 (8)	0.0161 (7)
C28	0.0567 (8)	0.0461 (8)	0.0746 (10)	−0.0121 (6)	0.0074 (7)	0.0004 (7)
C29	0.0444 (7)	0.0508 (8)	0.0735 (9)	−0.0006 (6)	−0.0038 (7)	−0.0107 (7)
C30	0.0461 (7)	0.0451 (7)	0.0506 (7)	0.0074 (6)	−0.0005 (6)	−0.0016 (6)
C31	0.0488 (7)	0.0432 (6)	0.0402 (6)	−0.0043 (5)	0.0044 (5)	−0.0006 (5)
C32	0.0629 (9)	0.0462 (8)	0.0618 (9)	0.0035 (6)	0.0119 (7)	0.0038 (7)
C33	0.0829 (11)	0.0433 (8)	0.0762 (11)	−0.0127 (8)	0.0268 (9)	−0.0024 (7)
C34	0.0889 (12)	0.0621 (10)	0.0641 (10)	−0.0392 (9)	0.0133 (9)	−0.0073 (8)
C35	0.0662 (10)	0.0737 (11)	0.0635 (10)	−0.0264 (8)	−0.0110 (8)	0.0130 (8)
C36	0.0544 (8)	0.0462 (7)	0.0566 (8)	−0.0089 (6)	−0.0034 (6)	0.0088 (6)

### Geometric parameters (Å, º)

**Table:**

F1—C15	1.3607 (15)	C17—H17	0.9500
F2—C22	1.3569 (15)	C18—C19	1.4858 (17)
O1—C2	1.3848 (15)	C19—C24	1.3899 (17)
O1—C25	1.3902 (14)	C19—C20	1.3928 (17)
O2—C8	1.3863 (14)	C20—C21	1.3810 (19)
O2—C31	1.3959 (15)	C20—H20	0.9500
O3—C11	1.2198 (14)	C21—C22	1.365 (2)
O4—C18	1.2144 (14)	C21—H21	0.9500
C1—C2	1.3788 (16)	C22—C23	1.369 (2)
C1—C10	1.4343 (17)	C23—C24	1.3854 (19)
C1—C11	1.5069 (16)	C23—H23	0.9500
C2—C3	1.4084 (17)	C24—H24	0.9500
C3—C4	1.3534 (19)	C25—C26	1.3776 (18)
C3—H3	0.9500	C25—C30	1.3815 (18)
C4—C5	1.4141 (17)	C26—C27	1.3872 (19)
C4—H4	0.9500	C26—H26	0.9500
C5—C6	1.4092 (18)	C27—C28	1.378 (2)
C5—C10	1.4326 (16)	C27—H27	0.9500
C6—C7	1.3605 (19)	C28—C29	1.377 (2)
C6—H6	0.9500	C28—H28	0.9500
C7—C8	1.4049 (17)	C29—C30	1.388 (2)
C7—H7	0.9500	C29—H29	0.9500
C8—C9	1.3781 (17)	C30—H30	0.9500
C9—C10	1.4322 (16)	C31—C36	1.3746 (19)
C9—C18	1.5084 (15)	C31—C32	1.3788 (19)
C11—C12	1.4846 (16)	C32—C33	1.375 (2)
C12—C13	1.3881 (17)	C32—H32	0.9500
C12—C17	1.3924 (18)	C33—C34	1.370 (3)
C13—C14	1.3869 (18)	C33—H33	0.9500
C13—H13	0.9500	C34—C35	1.384 (3)
C14—C15	1.365 (2)	C34—H34	0.9500
C14—H14	0.9500	C35—C36	1.389 (2)
C15—C16	1.367 (2)	C35—H35	0.9500
C16—C17	1.3815 (19)	C36—H36	0.9500
C16—H16	0.9500		
			
C2—O1—C25	119.09 (9)	C19—C18—C9	117.62 (9)
C8—O2—C31	117.61 (9)	C24—C19—C20	119.20 (12)
C2—C1—C10	119.33 (10)	C24—C19—C18	121.73 (11)
C2—C1—C11	116.65 (11)	C20—C19—C18	119.06 (11)
C10—C1—C11	123.72 (10)	C21—C20—C19	120.07 (13)
C1—C2—O1	117.02 (10)	C21—C20—H20	120.0
C1—C2—C3	122.63 (12)	C19—C20—H20	120.0
O1—C2—C3	120.00 (11)	C22—C21—C20	118.95 (13)
C4—C3—C2	118.69 (11)	C22—C21—H21	120.5
C4—C3—H3	120.7	C20—C21—H21	120.5
C2—C3—H3	120.7	F2—C22—C21	118.52 (14)
C3—C4—C5	121.83 (11)	F2—C22—C23	118.52 (15)
C3—C4—H4	119.1	C21—C22—C23	122.96 (13)
C5—C4—H4	119.1	C22—C23—C24	117.97 (14)
C6—C5—C4	120.04 (11)	C22—C23—H23	121.0
C6—C5—C10	120.15 (11)	C24—C23—H23	121.0
C4—C5—C10	119.80 (12)	C23—C24—C19	120.82 (13)
C7—C6—C5	121.72 (11)	C23—C24—H24	119.6
C7—C6—H6	119.1	C19—C24—H24	119.6
C5—C6—H6	119.1	C26—C25—C30	120.81 (12)
C6—C7—C8	118.50 (12)	C26—C25—O1	123.07 (11)
C6—C7—H7	120.8	C30—C25—O1	116.04 (11)
C8—C7—H7	120.8	C25—C26—C27	119.24 (13)
C9—C8—O2	116.03 (10)	C25—C26—H26	120.4
C9—C8—C7	122.67 (11)	C27—C26—H26	120.4
O2—C8—C7	121.09 (11)	C28—C27—C26	120.76 (14)
C8—C9—C10	119.64 (10)	C28—C27—H27	119.6
C8—C9—C18	115.79 (10)	C26—C27—H27	119.6
C10—C9—C18	124.36 (10)	C29—C28—C27	119.27 (13)
C9—C10—C5	117.31 (11)	C29—C28—H28	120.4
C9—C10—C1	124.99 (10)	C27—C28—H28	120.4
C5—C10—C1	117.66 (10)	C28—C29—C30	120.85 (13)
O3—C11—C12	121.69 (11)	C28—C29—H29	119.6
O3—C11—C1	119.31 (10)	C30—C29—H29	119.6
C12—C11—C1	118.97 (10)	C25—C30—C29	119.05 (13)
C13—C12—C17	119.14 (11)	C25—C30—H30	120.5
C13—C12—C11	121.70 (11)	C29—C30—H30	120.5
C17—C12—C11	119.16 (11)	C36—C31—C32	121.67 (13)
C14—C13—C12	120.66 (12)	C36—C31—O2	117.97 (12)
C14—C13—H13	119.7	C32—C31—O2	120.34 (12)
C12—C13—H13	119.7	C33—C32—C31	119.06 (15)
C15—C14—C13	118.02 (13)	C33—C32—H32	120.5
C15—C14—H14	121.0	C31—C32—H32	120.5
C13—C14—H14	121.0	C34—C33—C32	120.42 (15)
F1—C15—C14	118.62 (13)	C34—C33—H33	119.8
F1—C15—C16	117.99 (13)	C32—C33—H33	119.8
C14—C15—C16	123.39 (13)	C33—C34—C35	120.23 (15)
C15—C16—C17	118.26 (13)	C33—C34—H34	119.9
C15—C16—H16	120.9	C35—C34—H34	119.9
C17—C16—H16	120.9	C34—C35—C36	120.01 (16)
C16—C17—C12	120.52 (12)	C34—C35—H35	120.0
C16—C17—H17	119.7	C36—C35—H35	120.0
C12—C17—H17	119.7	C31—C36—C35	118.55 (14)
O4—C18—C19	122.34 (11)	C31—C36—H36	120.7
O4—C18—C9	119.96 (11)	C35—C36—H36	120.7
			
C10—C1—C2—O1	−174.65 (9)	C13—C14—C15—F1	179.56 (13)
C11—C1—C2—O1	−0.77 (15)	C13—C14—C15—C16	−1.0 (2)
C10—C1—C2—C3	−1.47 (17)	F1—C15—C16—C17	−179.61 (13)
C11—C1—C2—C3	172.42 (10)	C14—C15—C16—C17	0.9 (2)
C25—O1—C2—C1	−137.78 (11)	C15—C16—C17—C12	0.2 (2)
C25—O1—C2—C3	48.85 (14)	C13—C12—C17—C16	−1.10 (19)
C1—C2—C3—C4	2.12 (18)	C11—C12—C17—C16	178.19 (12)
O1—C2—C3—C4	175.10 (10)	C8—C9—C18—O4	−109.42 (13)
C2—C3—C4—C5	−0.34 (18)	C10—C9—C18—O4	65.25 (16)
C3—C4—C5—C6	178.22 (11)	C8—C9—C18—C19	67.27 (14)
C3—C4—C5—C10	−1.97 (17)	C10—C9—C18—C19	−118.07 (12)
C4—C5—C6—C7	179.33 (11)	O4—C18—C19—C24	−165.81 (12)
C10—C5—C6—C7	−0.47 (17)	C9—C18—C19—C24	17.59 (17)
C5—C6—C7—C8	1.01 (18)	O4—C18—C19—C20	15.05 (18)
C31—O2—C8—C9	−161.51 (11)	C9—C18—C19—C20	−161.55 (11)
C31—O2—C8—C7	23.62 (16)	C24—C19—C20—C21	−0.71 (19)
C6—C7—C8—C9	−1.41 (18)	C18—C19—C20—C21	178.45 (11)
C6—C7—C8—O2	173.11 (11)	C19—C20—C21—C22	−0.5 (2)
O2—C8—C9—C10	−173.55 (10)	C20—C21—C22—F2	−178.51 (12)
C7—C8—C9—C10	1.23 (17)	C20—C21—C22—C23	1.4 (2)
O2—C8—C9—C18	1.38 (15)	F2—C22—C23—C24	178.84 (12)
C7—C8—C9—C18	176.16 (10)	C21—C22—C23—C24	−1.1 (2)
C8—C9—C10—C5	−0.63 (15)	C22—C23—C24—C19	−0.2 (2)
C18—C9—C10—C5	−175.11 (10)	C20—C19—C24—C23	1.0 (2)
C8—C9—C10—C1	177.13 (10)	C18—C19—C24—C23	−178.09 (12)
C18—C9—C10—C1	2.65 (17)	C2—O1—C25—C26	26.01 (17)
C6—C5—C10—C9	0.26 (15)	C2—O1—C25—C30	−157.08 (11)
C4—C5—C10—C9	−179.54 (10)	C30—C25—C26—C27	1.6 (2)
C6—C5—C10—C1	−177.66 (10)	O1—C25—C26—C27	178.34 (13)
C4—C5—C10—C1	2.53 (15)	C25—C26—C27—C28	−1.1 (2)
C2—C1—C10—C9	−178.62 (10)	C26—C27—C28—C29	−0.3 (2)
C11—C1—C10—C9	7.96 (17)	C27—C28—C29—C30	1.2 (2)
C2—C1—C10—C5	−0.86 (15)	C26—C25—C30—C29	−0.7 (2)
C11—C1—C10—C5	−174.29 (10)	O1—C25—C30—C29	−177.67 (12)
C2—C1—C11—O3	−114.60 (12)	C28—C29—C30—C25	−0.7 (2)
C10—C1—C11—O3	58.99 (15)	C8—O2—C31—C36	−117.82 (13)
C2—C1—C11—C12	63.22 (14)	C8—O2—C31—C32	63.82 (16)
C10—C1—C11—C12	−123.19 (12)	C36—C31—C32—C33	2.7 (2)
O3—C11—C12—C13	−164.21 (12)	O2—C31—C32—C33	−179.01 (12)
C1—C11—C12—C13	18.03 (17)	C31—C32—C33—C34	−1.1 (2)
O3—C11—C12—C17	16.52 (17)	C32—C33—C34—C35	−1.0 (2)
C1—C11—C12—C17	−161.25 (11)	C33—C34—C35—C36	1.6 (3)
C17—C12—C13—C14	1.1 (2)	C32—C31—C36—C35	−2.0 (2)
C11—C12—C13—C14	−178.23 (12)	O2—C31—C36—C35	179.61 (13)
C12—C13—C14—C15	0.0 (2)	C34—C35—C36—C31	−0.1 (2)

### Hydrogen-bond geometry (Å, º)

Cg is the centroid of the C31–C36 ring.

**Table:**

*D*—H···*A*	*D*—H	H···*A*	*D*···*A*	*D*—H···*A*
C6—H6···O3^i^	0.95	2.40	3.3477 (15)	172
C13—H13···*Cg*^ii^	0.95	2.87	3.6924 (15)	145

Symmetry codes: (i) *x*, −*y*+1, *z*−1/2; (ii) −*x*, *y*, −*z*+1/2.
